# Rotavirus infection activates the UPR but modulates its activity

**DOI:** 10.1186/1743-422X-8-359

**Published:** 2011-07-20

**Authors:** Jose Luis Zambrano, Khalil Ettayebi, Walid S Maaty, Nicholas R Faunce, Brian Bothner, Michele E Hardy

**Affiliations:** 1 Immunology and Infectious Diseases, Montana State University, Bozeman MT, 59718, USA; 2 Chemistry and Biochemistry, Montana State University, Bozeman MT, 59717, USA

## Abstract

**Background:**

Rotaviruses are known to modulate the innate antiviral defense response driven by IFN. The purpose of this study was to identify changes in the cellular proteome in response to rotavirus infection in the context of the IFN response. We also sought to identify proteins outside the IFN induction and signaling pathway that were modulated by rotavirus infection.

**Methods:**

2D-DIGE and image analysis were used to identify cellular proteins that changed in levels of expression in response to rotavirus infection, IFN treatment, or IFN treatment prior to infection. Immunofluorescence microscopy was used to determine the subcellular localization of proteins associated with the unfolded protein response (UPR).

**Results:**

The data show changes in the levels of multiple proteins associated with cellular stress in infected cells, including levels of ER chaperones GRP78 and GRP94. Further investigations showed that GRP78, GRP94 and other proteins with roles in the ER-initiated UPR including PERK, CHOP and GADD34, were localized to viroplasms in infected cells.

**Conclusions:**

Together the results suggest rotavirus infection activates the UPR, but modulates its effects by sequestering sensor, transcription factor, and effector proteins in viroplasms. The data consequently also suggest that viroplasms may directly or indirectly play a fundamental role in regulating signaling pathways associated with cellular defense responses.

## Background

Rotavirus infections cause life-threatening gastroenteritis in infants and young children, resulting in considerable morbidity and mortality worldwide. Repeated exposure to infectious virus ultimately results in a protective immune response, as reflected by two efficacious vaccines licensed for use in multiple countries [1]. Rotaviruses are members of the family *Reoviridae *and contain a segmented double-stranded RNA genome encapsidated by a triple-layered protein shell. The genome encodes six structural proteins (VP1-VP6) and six nonstructural proteins (NSP 1-NSP6). Virus replication is completely cytoplasmic, and replication and double-layered particle assembly occurs in perinuclear inclusions called viroplasms [2-4]. Viral dsRNA replication is carried out within these inclusions, and structural proteins VP1, VP2, VP3 and VP6 accumulate to form the double-layered capsid (DLP). The mechanism of viroplasm assembly is unknown, however NSP2 and NSP5 are required for formation as well as for the recruitment of viral proteins [2,3,5-7]. Assembly of triple-layered particles occurs through binding of double-layered particles to NSP4, which is an ER transmembrane receptor for budding particles [8,9]. As particles bud through the ER they acquire VP7, VP4 and a transient envelope that is removed prior to release from cells by a Ca^2+^-dependent mechanism that is not completely understood [10,11]. Virus then is released from the cell by incompletely defined mechanisms that may include release by non-classical vesicular transport [12] and/or virus release upon cell death by cell lysis.

The global cell response to rotavirus infection manifested by changes in gene expression has been studied primarily at the transcript level. Cuadras et al reported the first comprehensive analysis of the transcriptional response to rotavirus infection in CaCo-2 cells [13]. Changes in transcript abundance were related to genes associated with multiple cellular processes including proteins associated with cell structure, stress, transcription regulators, calcium regulators and the IFN response. Other narrower investigations reported expression of cytokine and chemokine profiles in rotavirus infected cells or cells treated with virus-like particles [14-16]. The first studies on changes in gene expression in rotavirus infected cells at the protein level were performed by Taylor et al [17] using 2D gel electrophoresis and MS/MS. This study identified two chaperone proteins, GRP78 (also known as BiP) and GRP94 that were up-regulated at the level of both mRNA and protein. GRP78 and GRP94 are ER resident chaperones that assist in protein folding and were subsequently shown to play roles in rotavirus morphogenesis [17,18]. GRP78 also functions in regulation of the ER sensors of cell stress, as described later. The purpose of this study was to identify changes in the cellular proteome in response to rotavirus infection, particularly those that occur in the context of the IFN response. The Type I IFN response to rotavirus infection is receiving increased attention following identification of the viral IFN antagonist NSP1. NSP1 functions in targeted proteasome-dependent degradation of interferon regulatory factors 3, 5 and 7, and F-box protein β-TrCP, the result of which at minimum, is down-regulation of expression of IFN and IFN-regulated genes [19-23]. Additional mechanisms of IFN antagonism are evident in the prevention of nuclear translocation of the p65 subunit of NFκB, and STAT1 and STAT2 [21,24]. How rotavirus infection may modulate other cell signaling pathways that also function in host defense is not known.

Several proteins were identified as differentially regulated by OSU infection, IFN treatment or both, including the ER chaperones GRP78 and GRP94. Interestingly, most of the proteins modulated by OSU showed decreased levels, and many of these were associated with cellular stress responses. These identifications led us to further analyze proteins associated with cell stress, specifically the unfolded protein response (UPR). The primary function of the UPR is to restore cell homeostasis under conditions of ER stress brought on by accumulation of unfolded or misfolded proteins [25,26]. The data presented here show the UPR is activated in rotavirus infected cells, but then likely is down-regulated due to redistribution of ER chaperones, sensors and effector proteins to viroplasms. Together the results suggest viroplasms may play a lead role in the manipulation of cellular processes, in addition to its known function in rotavirus morphogenesis.

## Methods

### Cells and virus

MA104 monkey kidney cells were maintained in M199 media (Mediatech) supplemented with 5% fetal bovine serum (FBS, Atlanta Biologicals). Virus stocks of rhesus rotavirus RRV and OSU were prepared and titered as previously described [20]. For infections, virus was treated with 10 μg/ml of TPCK-trypsin for 30 minutes at 37°C and then inoculated onto MA104 cell monolayers at the desired multiplicity of infection.

### Infections and IFN treatments

2D-Differential Gel Electrophoresis (DIGE) experiments were performed with OSU. MA104 cells were cultured to confluence in 12 10 cm culture plates. Six of the plates were treated with 10 ml of serum free M199 containing 400 U/ml of IFNα (R&D Systems). The remaining six plates were treated with serum-free M199 without IFN. The plates were incubated for 18 hours at 37°C. After 18 hrs, three plates from each treatment group were infected with OSU at a multiplicity of three pfu/cell in fresh media with or without IFN. The remaining three plates in each treatment group were mock treated with the original contents (serum free M199, or serum-free M199 containing 400 U/ml of IFN). All plates were incubated for six hours at 37°C. The experimental outline resulted in four treatment groups with three biological replicates in each group: 1) mock infected, no IFN; 2) mock infected, IFN treated; 3) infected, no IFN; and 4) infected, IFN treated.

Cells were harvested and washed three times with 10 ml of calcium/magnesium-free PBS (PBS-cmf) containing 1.0 mM NaVO_3_. Cells were collected by centrifugation for 10 minutes at 500 × *g*. After the final wash, the supernatant was discarded and the pellets were suspended in 300 μl of 2-D gel sample buffer (30 mM Tris-HCl pH 8.5, 7 M urea, 2 M thiourea, 4% CHAPS, 1% ASB-14, 50 mM DTT, 0.002% bromophenol blue and protease inhibitor cocktail) and transferred to a 2.0 ml microcentrifuge tube. Acetone-precipitated proteins were collected by centrifugation for 30 minutes at 16,000 × *g *at 0°C. Pellets were suspended in 500 μl of 2D sample buffer and protein concentration was determined with the RcDc Protein Assay system (BioRad).

### CyDye Labeling

Proteins in each sample were labeled with CyDyes (GE Amersham) following the specifications of the manufacturer. The pH of the samples was adjusted to 8.5 by addition of 2D sample buffer containing 30 mM Tris, pH 8.5, and protein concentrations were determined by RcDc assay. CyDyes were reconstituted in DMF (Sigma: St. Louis, MO) according to the manufacturer's protocol. 50 μg of protein from each biological replicate in each treatment group were labeled with 400 pmol of CyDyes and incubated for 10 minutes on ice in the dark. The labeling reaction was stopped by the addition of 1.0 μl of 10 mM lysine, followed by a ten minute incubation on ice in the dark. Groups 1 and 3 were labeled with Cy3, and groups 2 and 4 were labeled with Cy5. 25 μg of protein from each of the 12 samples were pooled and batch labeled with 2400 pmol of Cy2 as the internal standard. Groups 1 and 2 were multiplexed on the same gels, and groups 3 and 4 were multiplexed on the same gels. 50 μg of the Cy2 labeled samples were added to each gel as the internal standard.

### 2D electrophoresis

24 cm IPG strips (pH 5.3-6.5 or pH 3-5.6, GE Amersham) were actively rehydrated for 20 hours at 50 volts in a Protean IEF cell (BioRad). Narrow range pH strips were used to increase the resolution. The rehydrated strips then were transferred to an IPGphor (GE Amersham) and proteins were focused at 20°C. Strips were equilibrated for 15 minutes in SDS equilibration buffer (50 mM tris-HCl pH 8.8, 6 M urea, 30 v/v glycerol, 2% w/v SDS, and .002% bromophenol blue) containing 65 mM DTT. The strips then were transferred to fresh SDS equilibration buffer containing 135 mM iodoacetamide and incubated for an additional 15 minutes. Second dimension separation was done in a DALT2 separation unit on a SDS-12% polyacrylamide gel sealed with 0.5% agarose.

### Image Analysis

Analytical gels were scanned at a resolution of 100 microns using the Typhoon imaging system (GE Amersham). Scanned images were analyzed with the Progenesis SameSpots software package (Nonlinear Dynamics). Spots were determined to be differentially up- or down-regulated based on both an ANOVA analysis and power determination between the normalized volumes of the spots from the averaged gel images for the four treatment groups. The threshold of significance was set to ANOVA p < 0.05 and a power value > 0.8. Spots that met both these statistical criteria were considered differentially regulated.

### Preparative gels, trypsin digestion and mass spectrophotometric (MS) analysis

Preparative gels were loaded with 500 μg to 1.0 mg of unlabeled protein in 2D focusing buffer (7 M urea, 2 M thiourea, 2% CHAPS, 1.5% pH 5.5-6.7 or pH 3.5-5.0 IPG buffer, 5 mM of fresh DTT, .002% bromophenol blue) in a final volume of 450 μl. The spots corresponding to those on the gel images from the Progenesis analysis were excised with a pipette tip, destained in 50% acetonitrile (ACN) in 50 mM NH_4_HCO_3 _and then dehydrated in a speedvac. Gel pieces were rehydrated in 100 μl of 1.5 mg/ml DTT in 25 mM NH_4_HCO_3 _for one hour at 56°C. The DTT solution was removed and replaced with 100 μl of 10 mg/ml iodoacetamide in 25 mM NH_4_HCO_3_. The tubes were gently shaken for 45 minutes on a vortex mixer at room temperature. The liquid was discarded and the gel pieces were washed with 100 μl of 100 mM NH_4_HCO_3 _with gentle shaking for 10 minutes at room temperature, washed twice more with 50% ACN/50 mM NH_4_HCO_3_, and then dehydrated for 15 minutes in a speedvac.

In-gel trypsin digestions for MS were performed as previously described [27]. Peptide fragments were loaded on a nanoC18 trap column and separated on a nanoC19 analytical column. Gradient elution was accomplished over 12 minutes at a flow rate of 0.5 μl/ml using Agilent's ChipCube LC module interfaced to an Agilent XCTUltra nanoESI-IonTrap-MS equipped with collision induced dissociation cell with Helium as the collision gas.

### Protein identification and Gene Ontology (GO) analysis

Peptides were identified by searching the NCBInr database with the Mascot search engine's MS/MS Ion Search (Matrix Science, http://www.matrixscience.com/). Carbamidomethylation was set as a fixed modification. Peptide tolerance was set a ± 0.8 Da and MS/MS tolerance to ± 0.3 Da. Only peptides that were determined to be statistically significant based on Mascot MOWSE score were considered for protein identifications. In the case of VP6 where only a single peptide was found, this peptide was consistently found in replicate runs and the MS/MS data was manually inspected.

GO analysis was performed with the Database for Annotation, Visualization and Integrated Discovery (DAVID; http://david.abcc.ncifcrf.gov/[28]. The entire gene list was subjected to Functional Annotation Clustering Tool with Homo sapiens as the background list. Annotation clusters with enrichment values over 1.8 (where < 1.3 is considered insignificant) were further considered.

### Immunoblots

Sixty μg of protein were loaded onto a 12% polyacrylamide gel and then proteins were transferred to nitrocellulose membrane. The membrane was blocked in 10% non-fat dry milk (BLOTTO) in PBS for 30 minutes, and then incubated with rabbit polyclonal anti-GRP78 antibody (Cell Signaling), followed by secondary HRP-conjugated goat-anti-rabbit antibody (Jackson ImmunoResearch). Proteins were detected with ECL chemiluminescent reagent (Thermo Scientific). Membranes were reprobed with mouse-anti-actin antibody (Abcam) as a loading control.

### Immunofluorescence microscopy

MA104 cells were cultured on coverslips in 24-well plates at a density of 2.5 × 10^5 ^cells/ml. At 48 h post seeding, cells were mock infected or rotavirus infected with OSU or RRV at a MOI of 5 pfu/cell. Seven hours post-infection (hpi) the cells were fixed with 4% paraformaldehyde (PFA) in PBS for seven minutes at room temperature (RT). Autofluorescent aldehyde groups were blocked with 50 mM ammonium chloride (NH_4_Cl) in PBS for 15 minutes at RT. Cells were permeabilized with 0.1% Triton X-100 in PBS for seven minutes, and then incubated with 3% bovine serum albumin (BSA) for one hour. The cells were labeled with specific antibodies for: GRP94 (Goat, Santa Cruz Biotechnologies), GRP78 (Goat, Santa Cruz Biotechnologies), ATF6 (Rabbit, AbCam), XBP1 (Mouse, Cell Signaling), p-PERK (Rabbit, Santa Cruz Biotechnologies), GADD34 (Goat, AbCam), Nrf2 (Rabbit, Santa Cruz Biotechnologies), and CHOP (Mouse, Cell Signaling). The secondary antibodies used were: Alexa 488 (Mouse-Rabbit Invitrogen), Alexa 594 (Mouse-Rabbit, Invitrogen) and FITC (Goat, Pierce) conjugated. Anti-rotavirus antibodies included anti-NSP1 (Rabbit) and VP6 (Mouse, 4B2D2). Antibodies to cellular proteins produced in rabbits were confirmed to have no reactivity to rotavirus proteins. Samples were mounted and sealed in anti-fade mounting medium ProLong Gold with DAPI (Invitrogen). All samples were observed with the epi-fluorescence microscope Eclipse 80i (Nikon), using an APO series lens 60×/1.40 (oil immersion) (Nikon). The images were acquired using a monochrome camera DS-Qi1Mc (Nikon) controlled by the Nis-Element software (Nikon, ver. 3.10). Images were edited for brightness and contrast using the ImageJ software (NIH, ver. 10.2).

### 2-Deoxy-glucose treatment

2-deoxy-glucose (2DG) was used to activate the UPR according to the protocol established by Gaddameedhi et al [29]. Briefly, MA104 cells were grown on glass coverslips to confluence, and then treated with 10 mM 2DG for 48 hours. Medium was changed to MEM containing 10% FBS without 2DG fourteen hours prior to mock infection.

### RT-PCR

MA104 cells were infected with RRV or OSU for 7 h at a MOI of 10 pfu/cell. Total cellular RNA was isolated with modified Trizol extraction (TRI Reagent; Molecular Research Center, Inc; Ohio, USA) and RNeasy column cleanup (RNeasy Mini Kit; Qiagen). RNA integrity was assessed with Agilent 2100 Bioanalyzer and Agilent RNA 6000 Nano Reagents. cDNA was synthesized using 0.5 μg RNA in a 20 μL reaction mixture using the Quantitect Reverse Transcription Kit (Qiagen) and Mastercycler Personal thermal cycler (Eppendorf). The primers specific to XBP1 (F-5'-AATGAAGTGAGGCCAGTGG-3'; R-5'-TCAATACCGCCAGAATCCATG-3') based on sequence accession NM_0050803 were purchased from Integrated DNA Technologies.

## Results

### Differentially expressed proteins in OSU infected and IFN treated cells

**Figure 1:**
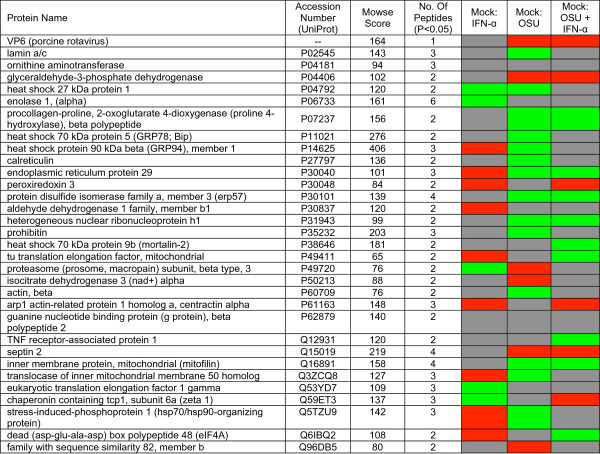
**Proteins differentially regulated in rotavirus infected and IFN treated cells**. Comparative changes in protein levels are shown in colored boxes. All changes are in comparison to mock-treated controls as indicated above the individual columns. Red indicates an increase, green indicates a decrease and gray indicates changes that were not statistically significant.

2D-DIGE is a sensitive and efficient method for screening a significant portion of the proteome for changes in protein expression. To improve the depth of coverage, narrow pH range isoelectric focusing (IEF) strips were used. Total soluble protein was analyzed using IEF ranges of 3.0-5.6 and 5.6-6.5, generating 495 and 950 protein spots on all replicate gels, respectively. Differential analysis showed that 123 spots were differentially regulated as defined by the statistical criteria outlined in Materials and Methods. Each of the 123 spots was selected for in-gel proteolysis and LCMS analysis, from which 32 unique protein IDs were returned (Figure 1). Nineteen proteins were modulated by OSU infection, with 13 of these showing decreased levels in infected cells compared to mock infected controls. The presence of up-regulated proteins without known internal ribosome entry sequences in their respective mRNAs suggests the observed decreases are not only a result of virus-induced global inhibition of cap-dependent translation. Additional evidence that translation of cellular mRNA still is occurring during infection is provided by detection of unfolded protein response effectors CHOP and GADD34 (see below).

The levels of 14 proteins were modulated by IFN treatment, and most showed an increase compared to mock treated controls. Fifteen proteins were differentially regulated when cells were treated with IFN prior to infection as compared to mock treated controls. Seven proteins of this group (not including VP6) were modulated by OSU infection alone, and the change in expression level was the same for each condition. That is, if a protein was down-regulated by IFN treatment prior to infection, it also was down-regulated during infection alone. These observations suggest OSU has little to no effect on this group of IFN-modulated proteins, which includes glyceraldehyde-3-phosphate dehydrogenase, proline-4-hydroxylase, erp29, erp57, hrnp H1, septin 2, and mitofilin. Six of the proteins differentially regulated in cells treated with IFN prior to infection also were modulated by IFN treatment alone. Erp29, Tu translation elongation factor (mitochondrial), and eIF4A were up-regulated by IFN treatment alone, but the levels were decreased in cells treated with IFN and then infected with OSU, suggesting virus infection may have a direct effect on expression of these proteins, even in the presence of IFN.

### GO analysis and co-regulated proteins

**Figure 2:**
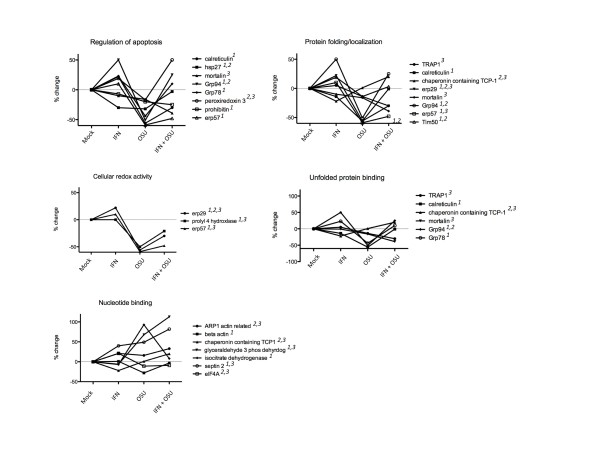
**Differential regulation of proteins identified by 2D-DIGE**. Percent change for each point is relative to mock controls. Numbers in italics in the graph legends refer to changes that achieved statistical significance under the condition of 1) OSU infection, *2*) IFN treatment and *3*) IFN treatment and OSU infection. Percent changes in other categories not indicated were not statistically significant, but trended toward increase or decrease.

GO analysis classified proteins into multiple cellular processes and functions including cellular redox activity, regulation of apoptosis, unfolded protein binding, nucleotide binding, protein folding and protein localization. Analysis of each of these categories in the context of levels associated with each of the treatment conditions suggests they are co-regulated upon IFN treatment and/or virus infection, as described below and as illustrated in Figure 2.

**Figure 3:**
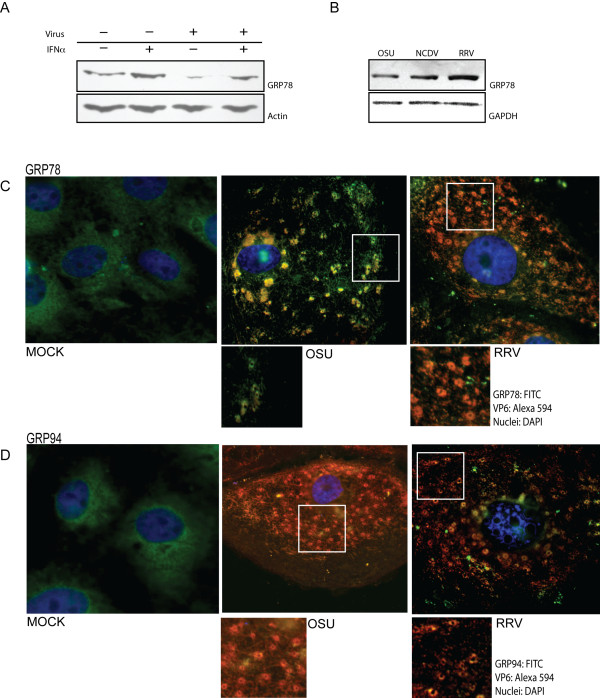
**Expression levels and subcellular localization of GRP78 and GRP94 proteins**. A) GRP78 expression levels in mock infected cells, OSU infected cells, IFN treated cells, or cells treated with IFN prior to infection as measured by immunoblot. 60 ug of protein from lysates prepared for 2D-DIGE were electrophoresed on SDS-polyacrylamide gels, and proteins were transferred to nitrocellulose. Membranes were probed with anti-GRP78 and anti-actin as a loading control. B) GRP78 expression in OSU, NCDV (bovine) and RRV-infected cells 7 hours post infection. C) GRP78 and D) GRP94 localization was performed in MA104 cells grown on glass coverslips. The cells were infected with OSU or RRV at a MOI of 5 pfu/cell. At 7 hpi, the cells were fixed stained with anti-GRP78 or anti-GRP94. Nuclei were labeled with DAPI.

A search with the GO term Biological Process returned 17 annotations (P < 0.01, Fisher's exact), segregated into two annotation clusters. Annotation cluster 1 included proteins with defined roles in apoptosis. Several showed increases or decreases when cells were treated with IFN, yet all showed decreased levels of expression upon virus infection compared to mock infected controls (Figure 2). Most showed trends towards increased levels when cells were pre-treated with IFN prior to infection. Five of the eight proteins with decreased levels of expression in OSU infected cells as compared to mock infected controls were classified as negative regulators of apoptosis, including HSP27, mortalin, GRP94, GRP78, peroxiredoxin 3, and Erp57, and are induced in response to cell stress. GRP78 was identified as down-regulated in OSU infected cells compared to mock infected controls in the proteomic analysis, and confirmed by immunoblot (Figure 3A). GRP78 is noted here because of previous data that demonstrate GRP78 is up-regulated in cells infected with rotavirus strain RRV, suggesting a potential difference in modulation of cellular responses between these two virus strains [17]. This difference was further confirmed by immunoblot (Figure 3B) showing a decreased amount of GRP78 in OSU infected cells compared to RRV infected cells or cells infected with bovine strain NCDV. Potential mechanisms explaining this difference are not currently understood but clearly reflect different host cell interactions that are dependent on virus strain. GRP94 also has been reported to be up-regulated in RRV infected cells [17], in contrast to the data reported here in OSU infected cells. GRP94 was up-regulated by IFN treatment, and GRP78 showed a trend toward increased levels, but was determined to be not significant.

GO Biological Process Annotation cluster 2 included proteins with roles in protein folding and localization (Figure 2). All but one of these proteins (chaperonin containing protein TCP-1) localize to the ER or to mitochondria. As before, protein levels either increased or decreased upon IFN treatment, with all but one (Erp29) decreased during virus infection. Many of these proteins function as molecular chaperones that generally are up-regulated in response to cell stress. The observation of decreased levels in OSU infected cells was somewhat surprising, but nonetheless consistent with a potential mechanism to down regulate the stress response that occurs non-specifically after viral infection.

GO Molecular Function returned 17 annotations (P < 0.05) and two annotation clusters. The first was associated with protein disulfide isomerase activity which functions in the oxidative environment of the ER to assist in disulfide bond formation and consequent protein folding. Erp29 and Erp57 were increased upon IFN treatment, but significantly down-regulated during virus infection. The second annotation cluster included proteins with roles in unfolded protein binding and nucleotide binding. Those involved in unfolded protein binding localize primarily to the ER and the mitochondria. As before, the levels of most of these proteins were decreased in OSU infected cells. In contrast to the results observed for proteins with roles in regulation of apoptosis, protein folding and localization, and unfolded protein binding, most of the proteins identified with functions that include nucleotide binding were increased upon OSU infection. The levels of these proteins, with the exception of β-actin, remained higher when cells were pre-treated with IFN.

#### Chaperones GRP78 and GRP94 localize to viroplasm patterns in infected cells

Apparent decreases in cell stress proteins and proteins associated with ER chaperone functions led us to further examine these pathways in rotavirus infected cells. Chaperones are responsible for correctly folding nascent proteins in the ER lumen prior to their translocation to appropriate subcellular locations. It has been suggested that GRP78, GRP94 or both, are involved in the morphogenesis of rotavirus [17]. More recently it was suggested that GRP94 protein may not be essential for virus replication, while GRP78 protein plays an active role in quality control in the assembly of mature rotavirus particles [18]. Both GRP78 and GRP94 were identified in the current study as differentially regulated by OSU infection, and thus we evaluated the subcellular localization of these proteins in infected cells. MA104 cells were infected with rotavirus strains OSU or RRV, and their localization was determined by immunofluorescence. In mock infected cells, GRP78 and GRP94 showed reticular and perinuclear staining, consistent with an ER distribution (Figures 3C and 3D). Changes in the distribution of GRP78 and GRP94 were observed in cells infected with either OSU or RRV. Both were redirected to the pattern of viroplasms, overlapping the staining pattern of viral protein VP6. These results are consistent with previous reports indicating that GRP94, as well as other chaperone proteins such as PDI (protein disulfide isomerase) and calreticulin changed their distribution in rotavirus infected cells to a pattern similar to viroplasms, but calnexin, another ER chaperone, did not [18]. We also found similar changes in the localization of proteins Erp57, PDI and calreticulin in infected cells; however, we also observed redistribution of calnexin protein in a similar pattern to that observed for the other chaperones (data not shown).

#### Proteins of the UPR redistribute to viroplasms in infected cells

Activation of the UPR results in increased expression of chaperones due to the activation of ER stress sensors ATF6 (activating transcription factor 6 [30,31], IRE1 (inositol requiring endonuclease 1) [32-34] and PERK (PKR-like ER kinase) [35,36]. The UPR sensors are transmembrane proteins with the lumenal domains bound to GRP78 [37]. Accumulation of unfolded proteins in the ER causes GRP78 to dissociate from the sensor proteins, leading to phosphorylation of IRE1 and PERK. ATF6 translocates to the Golgi where it is cleaved, and the transcriptionally active fragment is transported to the nucleus to bind promoters containing ER stress response elements [31,37].

**Figure 4:**
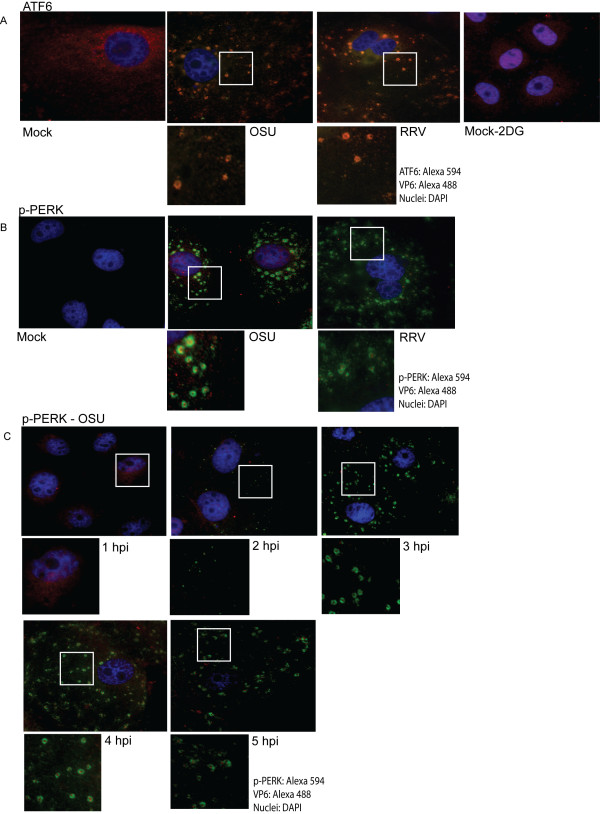
**Subcellular localization of UPR sensor proteins ATF6 and p-PERK**. MA104 cells were grown on glass coverslips and then infected with OSU or RRV at a MOI of five pfu/cell for seven hours. Mock infected cells treated with 2DG served as a positive control for UPR activation. A) ATF6 and B) p-PERK were stained with specific antibodies, followed by indicated Alexa-conjugated secondary antibodies. C) Epifluorescence microscopy of p-PERK expression over time during OSU infection (1-5 hpi) in MA104 cells.

The subcellular localization of ATF6 and thus activation of the UPR was determined in OSU and RRV infected MA104 cells seven hours post-infection. ATF6 localized to the ER in mock infected cells, and to the nucleus in cells treated with 2DG, which is known to activate the UPR [38] (Figure 4A). In contrast, ATF6 localization was similar to the staining pattern of VP6 in infected cells, suggesting that translocation to the nucleus was blocked. Likewise, phosphorylation of PERK (p-PERK) was evaluated by immunofluorescence under the same conditions. p-PERK was not detected in mock infected cells as expected. In infected cells, p-PERK was observed surrounded by VP6 in a pattern distinct from that of ATF6, GRP78 and GRP94 (Figure 4B).

A time course of infection was performed to determine at which stage of rotavirus replication the observed changes in the localization of p-PERK occurred (Figure 4C). p-PERK was detected in a reticular location very close to the nuclei at one hpi in cells infected with either OSU or RRV. At two hpi, VP6 began to accumulate in the cytoplasm while p-PERK persisted in the ER. However, at three hpi, p-PERK was observed in the viroplasm pattern, and continued to accumulate here between four and five hpi. These results suggest PERK is activated during the initial stages of rotavirus infection, but like ATF6, is redistributed to viroplasms, although as indicated above, in a distinctive pattern to suggest inclusion within viroplasms.

**Figure 5:**
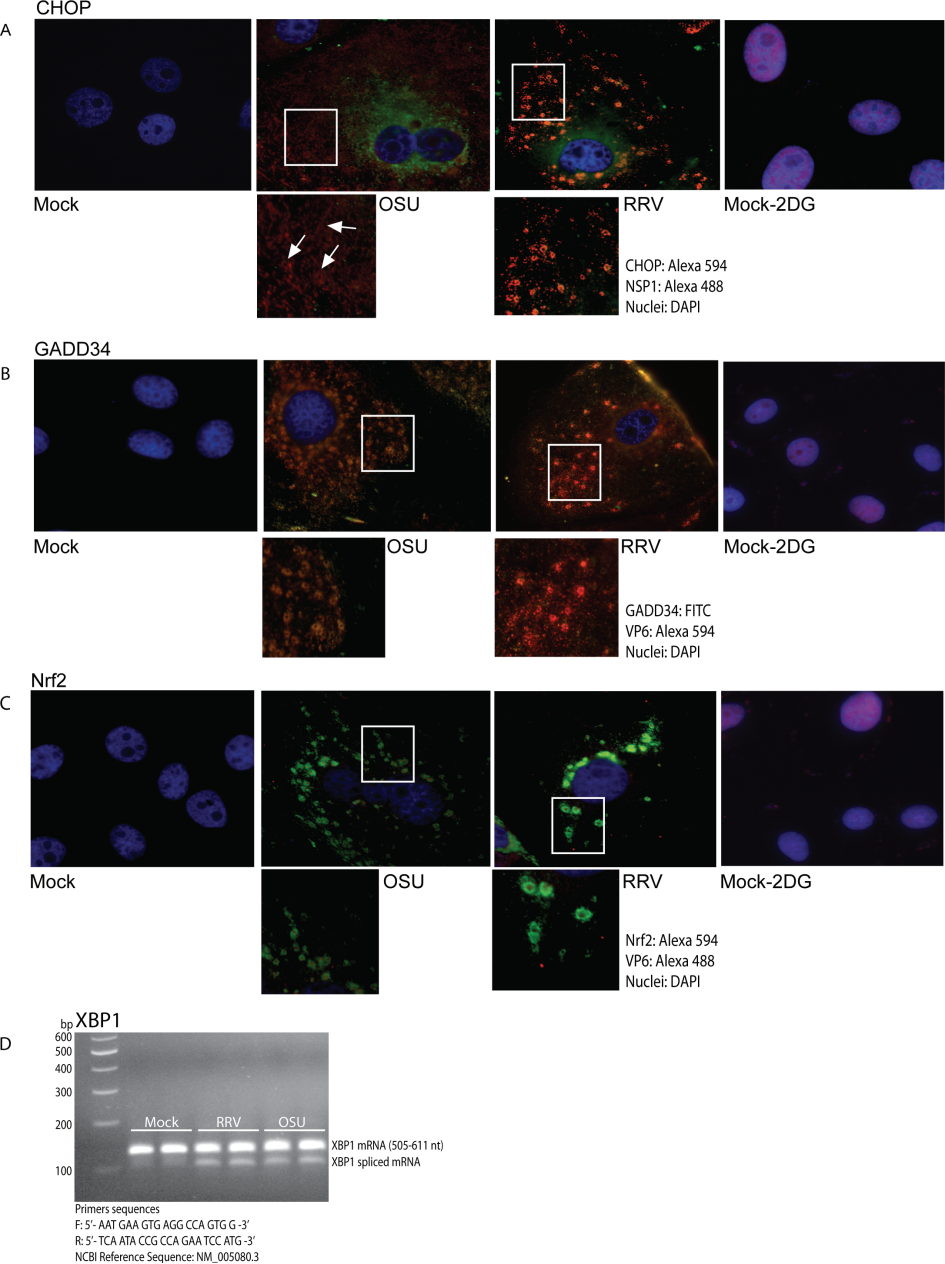
**Subcellular localization of transcription factors CHOP, GADD34 and Nrf2, and splicing of XBP1 mRNA**. MA104 cells were mock infected or infected with OSU or RRV for 7 hours at a MOI of 5 pfu/cell. A) CHOP, B) GADD34, and C) Nrf2. As a positive control for UPR activation, MA104 cells were treated with 10 mM 2DG for 48 hours prior to fixation. Nuclei were visualized using DAPI. Arrows in A indicate typical viroplasm localization of CHOP. D) MA104 cells were infected or mock infected, and RNA was extracted at seven hours post-infection and subjected to RT-PCR for detection of XBP1 mRNA. The presence of the doublet indicates the presence of the spliced form. Samples were run in duplicate.

Phosphorylation of IRE1 leads to splicing of XBP1 mRNA [34]. Translation of spliced XBP1 mRNA leads to synthesis of XBP1 protein that subsequently translocates to the nucleus to bind promoters containing ER stress response elements [39]. Although we could not detect phosphorylated IRE1 by immunostaining, RT-PCR for XBP-1 mRNA revealed the presence of both the spliced and unspliced form, suggesting that IRE1 was activated (Figure 5D). Similar to the distribution of chaperones and ATF6 in infected cells, XBP1 staining was consistent with the staining pattern of viroplasms, and its nuclear translocation thus was blocked (data not shown). Localization of UPR effectors CHOP and GADD34 that are induced by activation of the UPR also was similar to the pattern of viroplasms. These proteins are not expressed in mock-infected cells, suggesting that rotavirus infection induces expression of these proteins (Figures 5A, B).

## Discussion

We performed a small-scale proteomic analysis of cells infected with the OSU strain of rotavirus, cells treated with IFN, and cells treated with IFN prior to infection. The data described reflect differences in OSU-modulated, IFN-modulated and IFN pre-treatment-modulated proteins as compared to the mock controls. In general, trends were observed where identified proteins that increased upon IFN treatment were decreased in OSU infected cells. When cells were treated with IFN prior to infection, most proteins returned to basal levels and not to levels induced by IFN alone, suggesting some effect of virus replication on these proteins. More detailed analyses are needed to determine whether the proteins induced by IFN and then apparently controlled by OSU infection are truly part of a specific antiviral response.

While identification of stress response pathways is common in proteomics studies, proteins expected to increase were instead decreased upon OSU infection. This in contrast to what has been reported previously for rotavirus by others, likely explained by the differences in the rotavirus strains used for analysis. That there are differences in the basic virus-host cell interactions between rotavirus strains is illustrated by differences in the way they affect the host innate immune response. For example, RRV infection results in proteasome-dependent degradation of interferon regulatory factors 3, 5, and 7 [22,23], yet IRF3 is stable in OSU infected cells [21]. Thus it is conceivable that other strain-specific host cell interactions that occur that could be reflected in different cellular responses.

Several studies of rotavirus cellular pathogenesis have focused on perturbations of calcium homeostasis [10,40-44] and on alterations of cytoskeleton components [45-51], while others have investigated non-classic cellular mechanisms for viral protein transport [12,52,53]. Fewer studies exist on the impact of rotavirus infection on cell stress response pathways, and how rotavirus could modulate these pathways to attenuate innate defense mechanisms [21-23,54], including the ER-initiated UPR [18]. The ER plays a fundamental role in the morphogenesis of new rotavirus particles, as it is where final maturation of the particles occurs. ER calcium pools are reduced during rotavirus infection and cellular protein synthesis is redirected to favor viral protein synthesis [42,43]. Therefore, it is reasonable to predict that rotavirus infection leads to UPR activation. In this study, activation of the UPR sensors was demonstrated by detection of p-PERK, splicing of XBP1 mRNA suggesting IRE1 phosphorylation, and the redistribution of ATF6. In addition, expression of downstream effectors CHOP, GADD34 and Nrf2 (Figure 5C) also was detected. CHOP (GADD153) is a bZIP containing transcription factor, induced by ER stress, and over-expression of CHOP promotes apoptosis [55]. Like CHOP protein, GADD34 is expressed only under ER stress when PERK is phosphorylated [56]. Nrf2 is phosphorylated by PERK and translocates to the nucleus to activate transcription of antioxidant elements [57].

Phosphorylation of PERK leads to phosphorylation of translation initiation factor eIF2α [35,58,59] and it has been reported that eIF2α is phosphorylated in rotavirus infected cells by PKR [60]. These events trigger synthesis of transcription factor ATF4 that drives transcription of stress proteins Nrf2, GADD34 and CHOP, and the over-expression of chaperones including GRP78 [58]. We observed that Nrf2, GADD34, and CHOP are expressed in infected cells. However, the subcellular localization of these proteins was associated with viroplasm patterns and co-localized with VP6. All these proteins were surrounding the viroplasms, with the exception of Nrf2 that like p-PERK, appeared localized within viroplasms. In the early stages of infection with OSU or RRV (up to 2 hpi) p-PERK displayed an ER-cytoplasmic localization. At 3 hpi, p-PERK was observed surrounded by VP6, which increased further between 5 and 7 hpi. These results strongly suggest that in the early stages of rotavirus infection the PERK-dependent UPR pathway is efficiently activated. As the infection progresses, p-PERK is redirected to the viroplasms and sequestered into these viral structures, potentially avoiding amplification of the UPR. Inhibition of nuclear translocation of CHOP and GADD34, in addition to other UPR proteins, may serve to avoid the activation of cellular pro-apoptotic mechanisms. In a manner similar to initial activation of the UPR followed by down-regulation, Halasz et al. [61] reported activation of apoptosis in early stages of rotavirus infection in MA104 and HT29 cells, yet at six hours post-infection, markers of apoptosis including Annexin V and 7-AAD were absent. Our results show that pro-apoptotic transcription factors are expressed in early stages of rotavirus infection and then are directed or sequestered around or within viroplasms. Together, the data strongly suggest a loss of control of key metabolic pathways of signaling for an effective response against infection, and that viroplasms may play a role, directly or indirectly modulating cellular defense mechanisms.

As mentioned above, the phosphorylation of IRE1 leads the splicing of XBP1 mRNA, resulting in translation of XBP1. IRE1 phosphorylation also activates the cellular pathway of autophagy [62]. Like PERK-dependent stress proteins, XBP1 translocation to the nucleus was blocked and redistributed to viroplasms. We did not evaluate components of the autophagy pathway, however, it has been reported that LC3 a cellular marker of autophagy interacts with NSP4-EGFP [63]. Splicing of XBP1 mRNA and possible activation of autophagy suggests that like PERK, IRE1 is activated and sequestered by viroplasms at seven hpi.

Transcriptionally active ATF6 promotes expression of ER lumenal chaperones as well as CHOP and XBP1. The results shown in this study indicate that in a way similar to the other UPR sensors, ATF6 translocation to the nucleus is blocked at later times post infection, and instead colocalizes with VP6, again potentially affecting the efficiency of ATF6-dependent effector mechanisms. Repeated observations on the localization of the UPR proteins to follow the pattern of viroplasms may suggest that these viral inclusions may function not only in the morphogenesis of new virus particles, but also may play a central role in the evasion of cellular mechanisms that affect virus replication. This idea is further supported by previous observations that the p65 subunit of NFκB also localizes with viroplasms in OSU infected cells. Inhibition of nuclear translocation could be explained by the disorganization of the cytoskeleton reported during rotavirus infection. However, in cells infected with OSU, where translocation of p65 is inhibited, IRF3 accumulates in the nucleus, suggesting some selectivity to the inhibition process. Clearly the mechanisms by which rotavirus infection and the viral proteins involved in disrupting nuclear import of critical transcription factors need further exploration.

**Figure 6:**
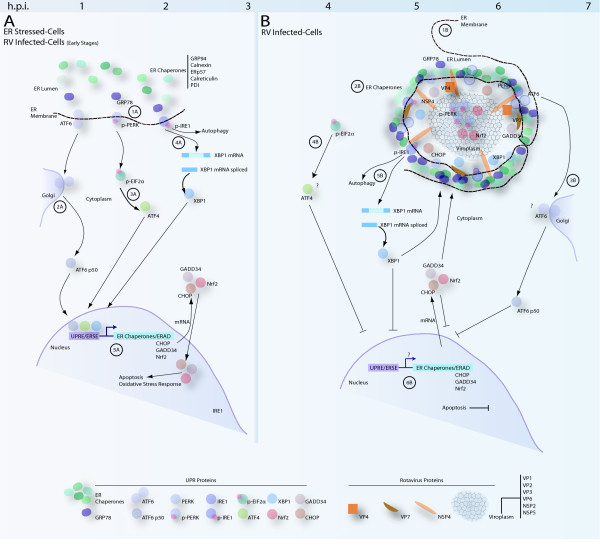
**Summary of the UPR pathway in rotavirus infected cells**. **6A**: 1A) Under ER stress, GRP78 is uncoupled from UPR sensors. 2A) ATF6 translocates to the Golgi where it is cleaved and transported to the nucleus. 3A) PERK is auto-phosphorylated. P-PERK phosphorylates eIF2α, stimulating the expression of ATF4, a transcription factor that is transported to the nucleus. 4A) IRE1 is auto-phosphorylated leading to splicing of XBP1 mRNA. XBP1 is transported to the nucleus. Phosphorylation of IRE1 may trigger autophagy via the JNK pathway. 5A) ATF6, ATF4 and XBP1 are bind to DNA upstream of UPR Element (UPRE) and ER Stress Element (ESRE) that triggers over-expression of other transcription factors and ER resident chaperones. Stress effectors proteins CHOP, GADD34, and Nrf2 may induce activation of other metabolic pathways, stress response or apoptosis. **6B: **1B) Viroplasms begin to assemble 3 hpi and interaction with the ER may induce morphological changes and rearrangement of the ER membrane. 2B) Chaperones are condensed around viroplasms. 3B) ATF6 is immobilized in the ER-viroplasms complex preventing its transport to the nucleus. 4B) p-PERK signal is sequestered within the viroplasms. 5B) XBP1 is expressed and directed to the ER-viroplasm complex. 6B) UPR effector proteins are expressed but their nuclear transport is blocked and instead they are sequestered in or around viroplasms.

Changes in the distribution of the UPR proteins observed in this study may be due to morphological changes that the ER must overcome during rotavirus infection rather than due to an activity modulated by viral infection. Electron microscopy studies have suggested that the ER cisternae envelop the viroplasms, providing one possible explanation for changes in the location of UPR proteins [53,64]. Redistribution of the UPR proteins may contribute to the recruitment of chaperones that are necessary for viral protein folding and particle assembly. The mechanism by which some cellular proteins become localized to viroplasms is not clear, and the interaction between viroplasms and ER membranes is not well defined. A summary of the activity of the UPR in rotavirus infected cells is provided in Figure 6.

We have shown by 2D-DIGE that several proteins associated with cell stress are decreased in OSU infected cells, and follow up studies indicate their subcellular redistribution in cells infected with either OSU or RRV. Together, the data indicate that the stress response, particularly the UPR is activated upon infection, but is prevented from amplifying by inhibition of nuclear translocation of key transcription factors, effector proteins, and redistributed chaperones. Further investigations are ongoing to ascertain a mechanism for sequestration of these proteins with viroplasms.

## Competing interests

The authors declare that they have no competing interests.

## Authors' contributions

JLZ designed UPR experiments, performed microscopy and wrote the manuscript. KE assisted in the proteomics studies, participated in experimental design and contributed to writing the manuscript. WSM assisted in the design, interpretation and performance of the proteomics experiments. NRF participated in experimental design, data interpretation, and performed RT-PCR. BB assisted in design and interpretation of the proteomics work. MEH conceived of the basic research premise, participated in experimental design and data interpretation, and assisted in writing the manuscript. All authors read and approved the final manuscript.
